# Systemic Infection from Purulent Vaginal Discharges

**Published:** 1876-05

**Authors:** 


Systemic Infection from Purulent Vaginal Discharges.—According to Dr. E. T. Easley, it is probable that septicæmia may supervene on a profuse and persistent leucorrhœa, and absorption in such cases takes place through the vaginal walls as well as other mucous surfaces. The reasons that lead him to this view are, in the first place, that endosmosis depends upon the attraction of the membrane for the liquids, and its current in mucous membranes is found, in accordance with their physiological action, to be from the exterior to the interior. The more minute and extensive the vascular supply of a membrane the larger, of course, will be the endosmotic surface presented. In the second place, the transudation in facility and rapidity bears an inverse ratio to the thickness of the epithelia; but thirdly, the fuller and more tense the vessels of a part are, the more difficult will be the process of absorption; and, conversely, a rapid movement in the capillary blood, with diminished tension of the vessels, greatly favors the reception into the circulation of matters fit for absorption. The vagina is among the most highly organized structures in the human economy; its vascular, lymphatic, and nervous connections being in immediate relation with those of the other pelvic viscera. This shows obviously the liability of extraneous matters from the vaginal walls being absorbed into the circulation, more particularly when these walls are in an unhealthy condition. It may be safely said, that a vaginitis attended with profuse yellow leucorrhœa, is certainly evidence of more or less extensive abrasion of the mucous epithelia of the genital canal; hence the readiness with which putrid or broken-down products may be taken up.—*Med. and Surg. Reporter.*

